# Low-dose methadone for refractory chronic migraine accompanied by medication-overuse headache: a prospective cohort study

**DOI:** 10.1007/s10072-020-04602-3

**Published:** 2020-07-20

**Authors:** Silvia Benemei, Chiara Lupi, Francesco De Cesaris, Niccolò Lombardi, Alessandra Bettiol, Alberto Chiarugi, Pierangelo Geppetti, Valentina Galli, Chiara Pracucci, Brunella Occupati, Guido Mannaioni

**Affiliations:** 1 Headache Center, Careggi University Hospital, Florence, Italy; 2 Department of Health Sciences, Headache Center, Careggi University Hospital, University of Florence, Florence, Italy; 3 Toxicology Unit, Careggi University Hospital, Florence, Italy; 4 NEUROFARBA Department, Toxicology Unit, Careggi University Hospital, University of Florence, Florence, Italy

**Keywords:** Clinical practice, Medication overuse, Methadone, Migraine, Prospective cohort study, Refractory headache

## Abstract

**Objectives:**

A refractory chronic migraine (RCM) accompanied by medication-overuse headache (MOH) is an extremely disabling disease. Evidence suggests that in selected patients, chronic opioids may be a valuable therapeutic option for RCM. The aim of the present study was to evaluate the effectiveness and safety of prophylaxis with low-dose methadone (LDM) in patients affected by RCM with continuous headache and MOH.

**Methods:**

A prospective cohort study was performed between May 2012 and November 2015 at the Headache Center and Toxicology Unit of the Careggi University Hospital. Eligible patients were treated with prophylactic LDM and followed up for 12 months. Headache exacerbations, pain intensity, use of rescue medications, and occurrence of adverse drug reactions (ADRs) were recorded.

**Results:**

Thirty patients (24 females, median age 48 years) were enrolled. Nineteen (63%) patients dropped out, mainly because of early ADRs (*n* = 10), including nausea, vomiting, and constipation. At last available follow-up, LDM was associated with a significant decrease in the number of headache attacks/month (from a median of 45 (interquartile range 30–150) to 16 (5–30), *p* < 0.001), in pain intensity (from 8.5 (8–9) to 5 (3–6), *p* < 0.001), and in the number of rescue medications consumed per month (from 95 (34–240) to 15 (3–28), *p* < 0.001). No misuse or diversion cases were observed.

**Conclusion:**

LDM could represent a valuable and effective option in selected patients affected by RCM with continuous headache and MOH, although the frequency of early ADRs poses major safety concerns. Randomized controlled trials are needed to confirm the efficacy and safety of LDM prophylaxis.

## Introduction

Chronic migraine is increasingly disabling, making patients with continuous headache the most disabled in the spectrum of chronic migraineurs [1]. Chronic migraine affects at least 1% of the general population [2], and refractoriness to treatments (RCM, refractory chronic migraine) [3] and medication-overuse headache (MOH) [1] often aggravate this condition. Even if there is no conclusive consensus on its definition, refractoriness is a clinically relevant phenomenon that refers to the failure of at least 2 of 4 prophylactic treatments of different pharmacological classes [4]. As the prophylaxis fails, the risk for the patient to experience medication overuse and, consequently, undergo MOH significantly increases. Importantly, even if the majority of patients who discontinued medication overuse substantially improve [5–8], drug discontinuation is not always sufficient for the reduction of headache attack frequency or intensity. In a considerable portion of patients, chronic pain and exposure to adverse drug reactions (ADRs), due to the intense consumption of nonsteroidal anti-inflammatory drugs (NSAIDs), analgesics, and/or triptans, lead to a vicious cycle that progressively deteriorates patients’ health and quality of life.

Despite some drawbacks, including the potential for misuse and diversion and the risk of cognitive impairment, continuous opioid therapy represents a reasonable prophylactic option for patients affected by RCM with continuous headache and MOH [9]. In 2009, the American Pain Society proposed chronic headaches as one of the four chronic pain conditions where continuous opioid therapy might be taken into consideration [10]. According to the data from longitudinal studies and a long-standing experience with refractory patients and opioid schedules, guidelines for the selection of patients eligible to continuous opioid therapy have been proposed [11].

Little evidence is available regarding the preferred opioid schedules for RCM with continuous headache and MOH. Methadone, because of the peculiar pharmacological profile of its racemic mixture of *(R)-* and *(S)-*isomers, seems to be a better candidate compared with other opioids, in particular for its duration of action (long-acting opioid), with an analgesic effect that persists for 4–6 h [12]. Methadone has a mean bioavailability of about 80%, which is much higher than for the other opioid; in addition, it also has a long terminal half-life ranging from 7 to 65 h compared with other clinically used opioids [13]. Although metabolism and disposition are highly variable among subjects, the appropriate dosage tailoring allows to maximally benefit of the pharmacokinetic profile of methadone for patients’ treatment. Its primary analgesic effect is mediated by the agonism of the sole *(R)-*methadone on μ-opioid receptors. However, both isomers act as antagonists of the N-methyl-D-aspartate (NMDA) glutamate receptor [14], likely contributing to a reduced opioid tolerance [15] especially at low doses [16, 17]. The favorable profile of methadone in terms of reduced tolerance in comparison with other opioids is another key point for the administration of methadone for chronic pain therapy. Some preclinical evidence suggests it has an optimal profile regarding the ability to induce opioid receptor internalization that may explain this clinical phenomenon [18]. Despite its proven efficacy, methadone has a relevant potential for drug interactions and may be associated with serious ADRs, among which is the dose-independent prolongation of the QT interval [19] leading to rare but potentially fatal arrhythmias. Thus, the clinical use of methadone requires trained physicians, a careful education of patients, and a strict monitoring; nevertheless, the use of low dosages is recommended.

In this clinical context, the aim of the present study was to evaluate the effectiveness and safety, at 12 months of treatment, of low doses of methadone (LDM) in patients affected by RCM with continuous headache and MOH.

## Methods

### Study design and setting

A prospective cohort study was performed at the Headache Center and Toxicology Unit of the Careggi University Hospital. The study was approved by the Ethics Committee on Clinical Research (*Comitato Etico Regione Toscana, Sezione Area Vasta Centro; approval number 6078*) and registered in the Italian Registry for Observational Studies held by the Italian Medicines Agency (AIFA). The study was performed following all the guidelines for observational studies with human subjects required by the institution with which all the authors are affiliated. A written informed consent for research was obtained from all participants.

### Study population

Between May 2012 and November 2015, patients aged ≥ 30 years were screened at the Headache Center of Careggi University Hospital and, if diagnosed with RCM with continuous headache and MOH, were informed about the possibility of receiving prophylactic LDM. Only patients with refractory headache or with contraindications  to the use of evidence-based interventions were eligible [11]. Patients were considered not eligible in case of contraindications to opioid treatment, including past addictive disease or serious mental illnesses [11]. Before starting the LDM, patients underwent a psychiatric evaluation in order to exclude lifetime diagnosis of schizophrenia or other psychiatric syndromes according to the Diagnostic and Statistical Manual of Mental Disorders (DSM) [5] and clinical assessment, including electrocardiography (ECG) and urine drug screening. According to standard clinical practice, patients that resulted eligible after clinical screening were informed about possible drug-drug interactions related to methadone treatment. In order to avoid possible interactions and optimize treatment, an informative letter was sent to the general practitioner of each patient.

According to standard practice, patients should have their headache diary reporting data about their chronic migraine in the previous 3 months. Information reported in this diary included the number of rescue medications (including non-opioid analgesics, NSAIDs, and triptans) consumed per month, the number of days with headache per month, the number of headache exacerbations deserving pain relievers per month, and their intensity assessed every day by the visual analogue scale (VAS).

Data from the headache diaries were used as baseline values for study assessments. After signature of the informed consent, eligible patients were transferred to the Toxicology Unit, where all the clinical procedures and study follow-up visits were performed.

The baseline visit was conducted at *T*_*0*_ (i.e., day of LDM start). All treated patients were prescribed with standard prophylaxis (i.e., enriched fiber diet, physical activity, lactulose if needed) to prevent methadone-induced constipation. LDM started from 2 mg per day and was increased or administered in multiple daily doses according to clinical evaluation, since no dosing strategy for initiation of therapy and later uptitration have been validated. In our patients, maximal dose administered was 30 mg per day at *T*_*0*_. and 40 mg per day at *T*_*4*_.

Follow-up visits were planned at *T*_*1*_ (30 days following *T*_*0*_), *T*_*2*_ (3 months after *T*_*0*_), *T*_*3*_ (6 months after *T*_*0*_), and *T*_*4*_ (12 months after *T*_*0*_). The variability in the time interval between subsequent visits was due to the increased need of follow-up in the initial phases of the treatment, when titration of the methadone doses can still be critical.

### Outcome evaluation

The primary outcome was headache exacerbations. The primary endpoint was the number of headache exacerbations per month requiring a pain reliever.

The secondary outcomes were the pain intensity and the need of rescue medications. The secondary endpoints were the changes in pain intensity, measured using the VAS, and the number of rescue medications consumed per month.

Safety outcomes included all ADRs occurred during LDM treatment. Namely, safety endpoints were the number and the grade of ADRs recorded. To this aim, ECG recordings were performed at *T*_*0*_, *T*_*1*_, and *T*_*4*_ in order to detect QTc changes possibly due to administration of LDM.

### Statistical analysis

Data were reported as mean value ± standard deviation of the mean (SD) or as median value and related interquartile range (IQR), according to data distribution.

Effectiveness and safety endpoints were evaluated at *T*_*1*_, *T*_*2*_, *T*_*3*_, and *T*_*4*_ and compared with *T*_*0*_ using the Wilcoxon test for paired data. Furthermore, effectiveness and safety endpoints at *T*_*0*_ were compared with those obtained at last available follow-up, i.e., *T*_*1*_, *T*_*2*_, *T*_*3*_, or *T*_*4*_, according to patients’ data availability. Statistical significance was considered for *p* value < 0.05. An analysis was conducted using the software STATA version 14.

## Results

Thirty patients were considered eligible for LDM and were further enrolled in the study. Of them, 24 were females (80.0%), with a median age of 48 years (41.2–54.2). Demographic and clinical characteristics of the enrolled cohort are detailed in Table 1**.**

**Table 1:** Demographic and clinical data of patients treated with low-dose methadone (LDM)

	Median (IQR) or n (%)
Demographic data	
Age (median years, IQR)	48 (41.2–54.2)^#^
Female	24 (80.0)
Overused drug/s*	
NSAIDs	28 (93.3)
Triptans	17 (56.7)
Opioids	17 (56.7)
Acetaminophen	11 (36.7)
Comorbidities	
Anxiety	20 (66.7)
Arterial hypertension	9 (30.0)
Other pain conditions	7 (23.3)

*Some patients overused more than one drug

*IQR* interquartile range, *NSAIDs* nonsteroidal anti-inflammatory drugs

Focusing on the previous pharmacological treatment of headache, 93.3% of patients overused NSAIDs (*n* = 28), 17 (56.7%) overused triptans, and the other 17 overused opioids. Acetaminophen was overused by 11 patients (36.7%). Notably, patients assumed more than one drug class to treat headache exacerbations. Concerning failed prophylaxes, tricyclic antidepressants, calcium-channel blockers, and antiepileptics were reported as previous treatment by 73% of patients, while beta-blockers and onabotulinum toxin A by 67% of patients. Importantly, 40% of patients are reported to have been treated with at least four of the abovementioned drug classes, while 60% of patients have tried them all.

As for comorbidities, most patients suffered from anxiety (66.7%), whereas arterial hypertension and other pain conditions were reported in 30.0 and 23.3% of patients, respectively.

LDM was initiated during in-hospital stay (2–3 days) in 28 patients, while 2 patients started LDM in a day-hospital setting. An initial mean dose of methadone was 12 ± 4 mg (IQR 8–17 mg).

**Fig. 1:**
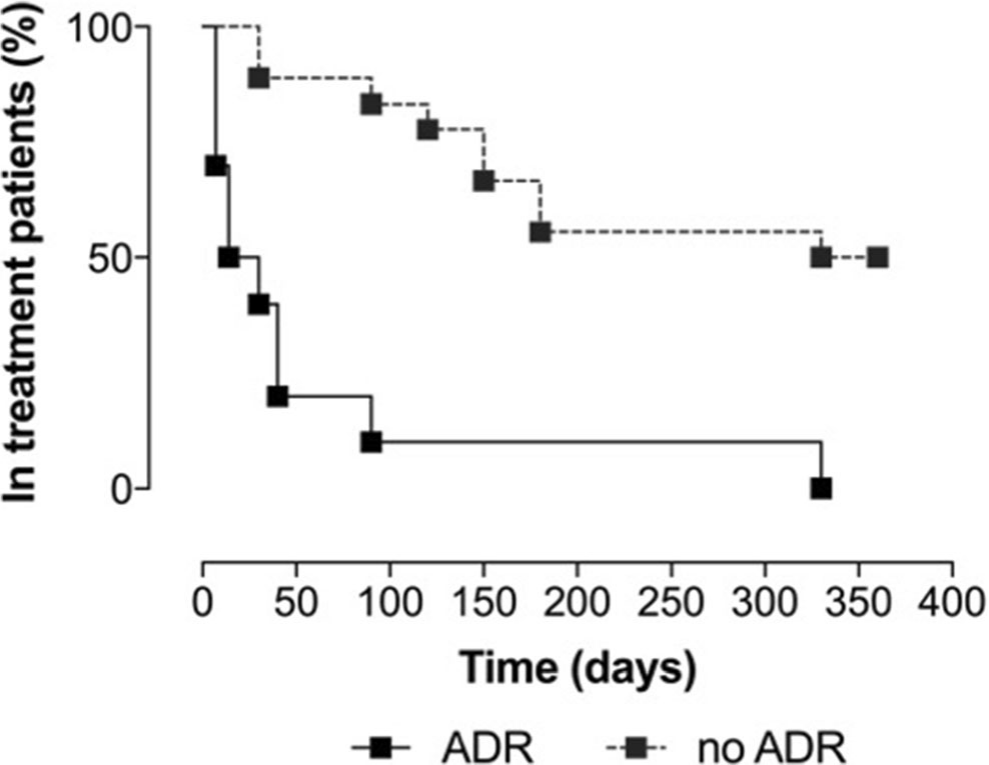
Time on treatment with low-dose methadone (LDM) of patients in 1 year of follow-up. Survival curves of patients that dropped out because of an ADR (continuous line) vs. all the other patients (still on treatment at month 12/dropouts for inefficacy/dropouts for personal reasons; dotted line). Importantly, most ADR patients dropped out early after LDM initiation (median time 14 days, IQ range 7–40). The curves are statistically different (log-rank Mantel-Cox test; *P* < 0.0001)

The demographic and clinical characteristics of patients are detailed in Table 2**.** Nineteen (63.3%) patients discontinued LDM. Specifically, five (16.7%) withdrew because of ineffectiveness, after a median time of 4.6 months (IQR 3.5–5.5). Although LDM treatment was effective, fourteen patients withdrew for other reasons. Among them, ten (33.3%) withdrew because of ADR; it is worth noting that all were female. Other three patients (10.0%) dropped out because of poor treatment confidence, while one patient moved to another country and was therefore lost to follow-up. Eleven patients (36.7%) were still on LDM treatment at the end of our study. The persistence on LDM treatment, distinguishing patients that developed an ADR (*n* = 10) from the others (*n* = 20), is shown in Fig. 1 (solid and dashed lines, respectively).

**Table 2:** Demographic and clinical data of patients treated with low-dose methadone (LDM), grouped according to clinical outcomes

	Patients with ongoing treatment	Dropout		
	*n* = 11	Inefficacy *n* = 5	ADRs n = 10	Others *n* = 4
	Median (IQR) or *n* (%)	Median (IQR) or *n* (%)	Median (IQR) or *n* (%)	Median (IQR) or *n* (%)
Demographic data				
Age (median years, IQR)	48, 41–58	47, 28–64.5	49, 47.5–56.7	41.5, 39.5–51.7
Female	8 (72.7%)	3 (60%)	10 (100%)	3 (75%)
Prevalent headache type				
Migraine	10 (90.9%)	4 (80%)	10 (100%)	2 (50%)
Tension-type headache	1 (9.1%)	0 (0%)	0 (0%)	2 (50%)
Cluster headache	0 (0%)	1 (20%)	0 (0%)	0 (0%)
Overused drug/s*				
NSAIDs	10 (90.9%)	4 (80%)	10 (100%)	4 (100%)
Opioids	8 (72.7%)	1 (20%)	4 (40%)	4 (100%)
Acetaminophen	5 (45.4%)	1 (20%)	4 (40%)	1 (25%)
Serotonin receptor agonists	5 (45.4%)	4 (80%)	6 (60%)	2 (50%)
Comorbidities^§^				
Anxiety	9 (81.8%)	2 (40%)	6 (60%)	3 (75%)
Arterial hypertension	3 (27.3%)	2 (40%)	4 (40%)	0 (0%)
Other pain conditions	3 (27.3%)	1 (20%)	2 (20%)	1 (25%)

*Some patients overused more than one drug. ^**§**^*Some patients have more than one concomitant disease in addition to RCM*

*ADRs* adverse drug reactions, *IQR* interquartile range, *NSAIDs* nonsteroidal anti-inflammatory drugs

**Fig. 2:**
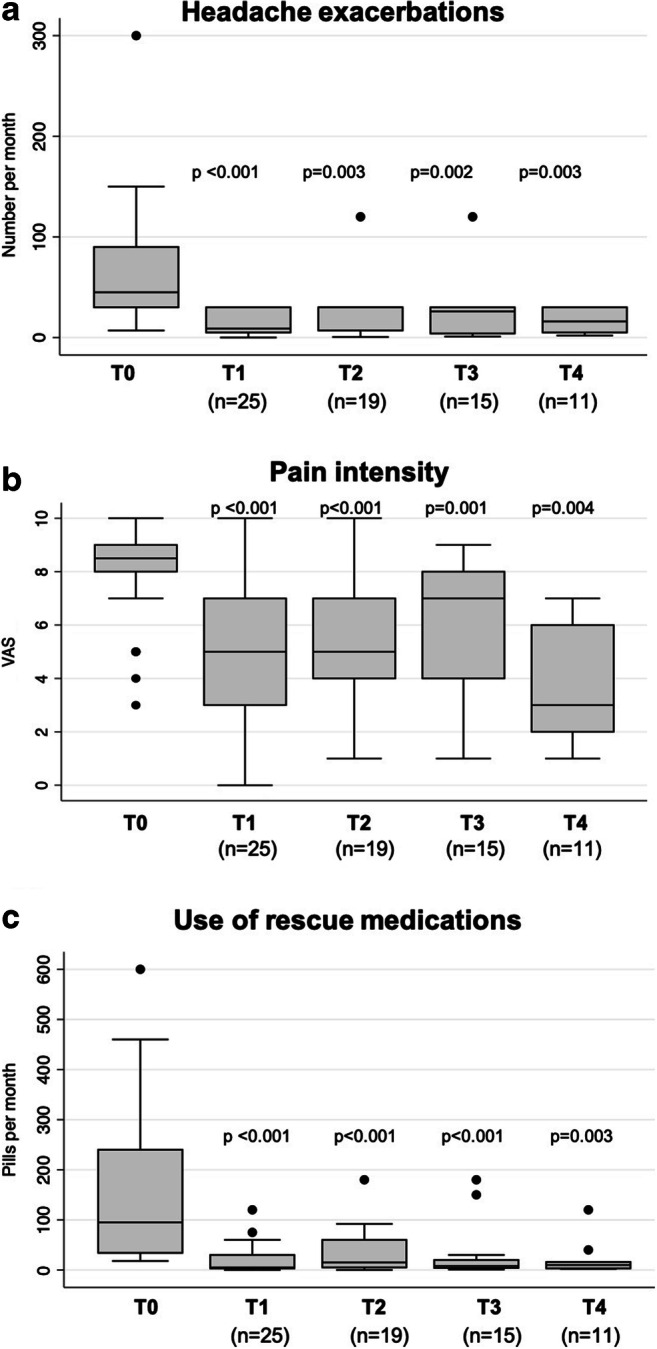
Effectiveness of low-dose methadone (LDM) on headache attacks, pain intensity, and the use of rescue medications. LDM decreased the number of headache exacerbations deserving a pain reliever per month (A), pain intensity assessed by a visual analogue scale (VAS) (B), and the number of pills of rescue medications consumed per month (C) at different time points of follow-up (*T*_*0*_ (baseline), *T*_*1*_ (30 days following *T*_*0*_), *T*_*2*_ (3 months after *T*_*0*_), *T*_*3*_ (6 months after *T*_*0*_), and *T*_*4*_ (12 months after *T*_*0*_). Namely, the median number (interquartile range, IQR) of headache exacerbations per month was 45 (30–90) at *T*_*0*,_ 9 (5–30) at *T*_*1*_, 30 (7–30) at *T*_*2*_, 26 (4–30) at *T*_*3*_, and 16 (5–30) at *T*_*4*_. The median VAS (IQR) was 8.5 (8–9) at *T*_*0*_, 5 (3–7) at *T*_*1*_, 5 (4–7) at *T*_*2*_, 7 (4–8) at *T*_*3*_, and 3 (2–6) at *T*_*4*_. The median number of pills of rescue medications (IQR) was 95 (34–240) at *T*_*0*_, 5 (3–30) at *T*_*1*_, 15 (5–60) at *T*_*2*_, 8 (4–20) at *T*_*3*_, and 10 (3–16) at *T*_*4*_

The effectiveness of LDM in terms of headache exacerbations, pain reduction, and use of rescue medications is described in Fig. 2. At time of start of LDM treatment (*T*_*0*_), the mean number of headache exacerbations per month requiring a pain reliever in the 30 enrolled patients was of 68.5 ± 60.4 (median 45, IQR 30–90) (Fig. 2A). At *T*_*1*_, the mean number of attacks requiring a pain reliever in the 25 observed patients significantly decreased to 15.9 ± 12.4 (median 9, IQR 5–30; *p* < 0.001). This significant reduction in the monthly number of headache exacerbations was confirmed also at the other time points of follow-up. Specifically, at *T*_*2*_, among the 19 patients for whom follow-up data were available, the mean number of attacks was of 24.8 ± 26.0 (median 30, IQR 7–30; *p* = 0.003). At *T*_*3*_, among the 15 observed patients, the mean of attacks was of 24.9 ± 29.2 (median 26, IQR 4–30; *p* = 0.002), and at *T*_*4*_, the mean number in the 11 observed patients was of 17.1 ± 11.4 (median 16, IQR 5–30; *p* = 0.003). At time of the last follow-up available for each patient (ranging from *T*_*1*_ to *T*_*4*_, *n* = 25), the mean number of headache attacks per month requiring a pain reliever was of 21.0 ± 23.6 (median 16, IQR 5–30, *p* < 0.001, *data not shown*).

Considering pain intensity, the mean VAS score at *T*_*0*_ in the 30 enrolled patients was of 8.0 ± 2.0 (median 8.5, IQR 8–9) (Fig. 2B). At *T*_*1*_ (25 observed patients), the mean VAS score significantly decreased to 5.0 ± 2.4 (median 5.0, IQR 3–7; *p* < 0.001). This significant reduction in pain intensity was confirmed also at *T*_*2*_ (19 patients; mean VAS of 5.4 ± 2.3; median 5, IQR 4–7; *p* < 0.001), *T*_*3*_ (15 patients; mean VAS of 5.9 ± 2.7; median 7, IQR 4–8; *p* = 0.001), and *T*_*4*_ (11 patients; mean VAS of 3.6 ± 2.3; median 3, IQR 2–6; *p* = 0.004). At time of the last follow-up available for each patient, the mean pain intensity was of 4.8 ± 2.3 (median 5, IQR 3–6, *p* < 0.001, *data not shown*).

A similar trend in pain relief was observed also considering the use of rescue medications (Fig. 2C). At *T*_*0*_, the mean number of pills used per month was of 163.3 ± 156.3 (median 95, IQR 34–240). At T_1_, the use of rescue medications significantly decreased to a mean of 21.3 ± 29.6 pills per month (median 5, IQR 3–30, *p* < 0.001). Similarly, at *T*_*2*_, *T*_*3*_, and *T*_*4*_, the monthly intake of rescue medications was reduced to a mean of number of 36.4 ± 45.6 pills (median 15, IQR 5–60, *p* < 0.001), 30.3 ± 55.5 pills (median 8, IQR 4–20, *p* < 0.001), and 21.8 ± 34.3 pills (median 10, IQR 3–16, *p* = 0.003), respectively. At time of the last follow-up, the mean number of pills used per month was of 22.2 ± 29.2 (median 15, IQR 3–28, *p* < 0.001, *data not shown*).

Ten patients reported clinically relevant ADRs (from low to moderate grade), requiring LDM discontinuation. Specifically, five patients had nausea, three vomiting, and two had constipation. Nine patients who experienced an ADR dropped out early after LDM initiation (after a median time of 14 days, IQ 7–40), while one patient dropped out at month 11. All patients fully recovered after tapered interruption of LDM. No other ADRs were observed in our sample. No case of misuse or diversion was observed (*data not shown*).

## Discussion

This is the first study evaluating the effectiveness and safety of LDM over a 12-month follow-up period, in patients affected by RCM with continuous headache and MOH in a real-world setting. As mentioned above, refractoriness may be diagnosed when a patient experiences ineffectiveness of at least 2 of 4 prophylactic treatments of different pharmacological classes [4]. Our results show that in patients affected by RCM, when tolerated, LDM is an effective option for the prevention of headache exacerbation, as well as for the reduction of pain intensity and consumption of rescue medications.

However, a significant portion of patients, despite an initial benefit from LDM, discontinued the treatment because of ADRs, although they were expected and non-serious. Even if the portion of patients developing nausea and vomiting was quite similar to that observed in other populations [20], our patients did not develop the expected tolerance to these ADRs. Accordingly, we cannot exclude that patients with migraine have a disease-related alteration (read as hypersensitivity) to these disturbances. This phenomenon, being nausea and vomiting the most frequent causes of LDM withdrawal in our population, deserves future investigation. Importantly, the median time to develop ADRs, being rather short (14 days) after therapy initiation, favored the safety profile as tapering of methadone was quick and easy. Altogether, our observations suggest that the optimization of the treatment, including either the association with or a formulation containing methylnaltrexone, which has been shown to be able to counteract the constipation [21], would relevantly increase the persistence on treatment, thus increasing the proportion of patients that might benefit from LDM. Another interesting result emerging from our population is that all patients experiencing ADR were female. An increased sensitivity to opioid-induced nausea and vomiting in women has been already reported [22–27], but the underlying mechanism is not known. In our study, the exclusive involvement of women may be due to the low number of participants that, however, per se discourages further subgroup analysis.

The impressive reduction of drug consumption observed in our study (from a median of 95 pills per month (IQR) (34–240) to 15 (3–28), *p* < 0.001) suggests that the initial medication overuse is mostly driven by the pain intensity. It is worth noting that this is at odds with the assumption that medication overuse in patients with migraine is mainly due to a genetic predisposition to substance abuse [28]. Indeed, when methadone is administered to patients with MOH according to a scheduled plan, it alleviates pain and drug consumption consequently falls. The fact that none of our patients misused methadone, notwithstanding its well-known abuse potential, further corroborates the hypothesis that RCM patients with MOH are not genetically predisposed drug abusers per se [28], but just deserve an efficacious pain treatment to defeat the vicious cycle that sustains medication overuse. However, as no conclusive evidence exists, it would be of paramount importance to dissect the mechanisms that drive the medication overuse in patients with migraine as this could significantly change the therapeutic approach to these patients.

It has been already reported that prescribing methadone for headache patients is neither glamorous nor lucrative and that it is a tedious process because of extensive patient education, controlled substance agreement, and meticulous record keeping [29]. However, the absence of suitable pharmacological alternatives for RCM associated with MOH is associated with an increased risk of ADRs due to overused medications and severe disability of patients who still seek medical attention after a number of therapeutic failures [29]. In this context, practitioners should consider LDM as a potential effective alternative for these patients. Currently, there are no restrictions for methadone prescriptions in Italy, since by the release of Law 38/2010 that specifically deals with the treatment of pain, it can be prescribed with the same modalities used for any other prescription drugs. Nonetheless, methadone prescription should still be reserved to specialists experienced in patients’ education and methadone handling. The opioid epidemics emerging in the United States, following to a well-meaning movement emerged in the United States 20 years ago to promote an adequate treatment of chronic pain with opioids and causing the death of almost half a million Americans from drug overdoses, suggests that more than caution is needed [30]. Despite the low rate of use of opioids in most European countries [31], the strict adherence to available guidelines that reserve opioids for headache patients only in selected cases [32] is both essential and mandatory to pursue an optimal management of LDM prophylaxis and to minimize the occurrence of overuse and ADRs.

Our results have several limitations. Unquestionably, the major limitation is the small sample size that is however due to the investigated condition. Considering the low frequency of the disease and the restrictedness of criteria that candidate a patient to the treatment, we had to deal with study premises resembling those of rare diseases, indeed. Although we planned a self-controlled study to maximize the internal validity of the study [33, 34], only initial evidence may emerge from observations in such a small population.

Another relevant drawback is the high rate of early treatment discontinuation, which significantly compromised the power of the study. It is worth noting that the study has a pragmatic approach and that the high portion of patients who discontinued the treatment represents the first valuable result of the study, indeed. On the one side, it suggests that only some weeks are needed to understand if patients will tolerate LDM. On the other side, as discontinuation was mainly due to gastrointestinal liability, we may hypothesize that innovative formulations of methadone will significantly decrease the number of patients who discontinue the treatment. Nevertheless, it is reasonable to consider that eligible enrolled patients were exclusively those non-responders to standard treatments, thus suggesting that LDM can represent a valid prophylactic approach in those refractory patients.

## Conclusions

LDM may be an effective prophylactic alternative for patients affected by RCM with continuous headache associated with MOH refractory to standard treatments. In eligible patients, after a short initial trial to test tolerability, LDM may represent a simple and inexpensive way to bring relief and reduce headache medication overuse, although the frequency of early ADRs poses major safety concerns. Further research, including assessment of methadone plasma levels and pharmacogenetic profiling, are needed to understand factors that may influence the clinical response to LDM in RCM patients. In addition, randomized controlled trials are needed to confirm the efficacy and safety of LDM prophylaxis.

## Data Availability

The datasets generated during and/or analyzed during the current study are available from the corresponding author on reasonable request.
